# Laser-Driven Ion Acceleration from Plasma Micro-Channel Targets

**DOI:** 10.1038/srep42666

**Published:** 2017-02-20

**Authors:** D. B. Zou, A. Pukhov, L. Q. Yi, H. B. Zhou, T. P. Yu, Y. Yin, F. Q. Shao

**Affiliations:** 1 College of Science, National University of Defense Technology, Changsha 410073, People’s Republic of China; 2 Institut für Theoretische Physik I, Heinrich-Heine-Universität Düsseldorf, Düsseldorf, 40225 Germany; 3 IFSA Collaborative Innovation Center, Shanghai Jiao Tong University, Shanghai 200240, People’s Republic of China

## Abstract

Efficient energy boost of the laser-accelerated ions is critical for their applications in biomedical and hadron research. Achiev-able energies continue to rise, with currently highest energies, allowing access to medical therapy energy windows. Here, a new regime of simultaneous acceleration of ~100 MeV protons and multi-100 MeV carbon-ions from plasma micro-channel targets is proposed by using a ~10^20^ W/cm^2^ modest intensity laser pulse. It is found that two trains of overdense electron bunches are dragged out from the micro-channel and effectively accelerated by the longitudinal electric-field excited in the plasma channel. With the optimized channel size, these “superponderomotive” energetic electrons can be focused on the front surface of the attached plastic substrate. The much intense sheath electric-field is formed on the rear side, leading to up to ~10-fold ionic energy increase compared to the simple planar geometry. The analytical prediction of the optimal channel size and ion maximum energies is derived, which shows good agreement with the particle-in-cell simulations.

Laser-driven energetic ion beam has attracted great attention because of its significance in many research communities and industrial applications. Compared with the conventional accelerators, these beams can be generated over only a few micrometer distances, having shorter pulse duration and more sufficient intensities. Over the past two decades, several mechanisms for laser-driven ion acceleration have been studied theoretically and experimentally, including target normal sheath acceleration (TNSA)^1^,^2^,^3^, radiation-pressure acceleration (RPA)^4^,^5^,^6^,^7^,^8^, breakout afterburner acceleration (BOA)^9^,^10^, and shock acceleration^11^,^12^. However, the maximum achieved energies^13^,^14^ of ~85 MeV for protons and ~20 MeV/u for carbon-ions are still unmatched for the demand of the particular applications. For the current laser facilities, TNSA has been identified as one of the most robust mechanisms. In this regime, the sheath acceleration electric field, scales as^15^ *E*_*sheath*_ ∝ (*n*_*h*_*T*_*h*_)^1/2^, is dependent on the hot-electron density *n*_*h*_ and temperature *T*_*h*_. For a simple planar target, *J* × *B* heating^16^ or vacuum heating^17^ dominates the hot-electron generation, while the obtained hot-electrons are usually *k*_*B*_*T*_*h*_ < *eϕ*_*p*_ and *n*_*h*_ < *n*_*c*_, in which *k*_*B*_ is the Boltzmann constant, *eϕ*_*p*_ is the ponderomotive potential and *n*_*c*_ is the critical plasma density^18^. Many methods such as placing suitable-scale preplasma^19^,^20^ and employing nanosphere surface^21^,^22^,^23^ or microcone^24^,^25^ has been suggested to heat the electrons. Nevertheless, simultaneously great increase of *n*_*h*_ and *T*_*h*_ remains a challenging endeavor. Recently, a micro-tube target has been identified to an usable method to achieve the light intensification for a *I* ≥ 10^22^ W/cm^2^ laser intensity and thus increase the electron temperature *T*_*h*_^26^. For *I* < 10^22^ W/cm^2^ being in the attainable domain of present laser conditions, it is found that the laser field is mainly depleted by the “dragged-out” electrons from the tube and loses the amplification effect^26^. The generated electron bunches, with extremely high densities and temperatures, are hopeful to act as externally injected hot-electron sources for TNSA and increase ionic energies dramatically.

In this article, we report on a considerable energy advancement of TNSA protons and carbon-ions by utilizing a ~10^20^ W/cm^2^ modest intensity laser incident on a plasma micro-channel target (CT). The CT structure is composed of a preposed gold micro-channel and an attached plastic substrate. With the aid of two-dimensional (2D) particle-in-cell (PIC) simulations, we find that the overdense (far beyond *n*_*c*_) electron sources with “superponderomotive” temperatures (*k*_*B*_*T*_*h*_ > *eϕ*_*p*_) are generated in the plasma channel. As the externally injected hot-electron sources, an intense sheath electric-field will be induced when they penetrate through the substrate, which sharply enhances ion acceleration. Hundreds of MeV protons and carbon-ions, almost one order of magnitude higher than that achieved using the usual planar target (PT), are simultaneously produced.

## Results

### Numerical and theoretical modelling

To explore the dynamic of the laser interacting with the plasma micro-channel target, we have carried out 2D PIC simulations with the collision and ionization effects included. A *p*-polarized planar wave, with *λ*_0_ = 0.8 *μ*m and *T*_0_ = 2.67 fs being the laser wavelength and period, is perpendicularly incident into the plasma micro-channel along the laser axis. The laser pulse has a temporal profile of 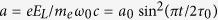, where *E*_*L*_ is the laser electric field, *a*_0_ = 10 is the normalized laser amplitude, and *τ*_0_ = 10*T*_0_ is the pulse duration. This corresponds to a laser intensity of ~2.14 × 10^20^ W/cm^2^, power of ~44 TW and total energy of ~1.2 J. The channel, of length *L*_0_ = 8.0*λ*_0_, wall-thickness *r*_0_ = 0.5*λ*_0_ and transverse interval *d*_0_ = 3.0*λ*_0_, is located between *x*_0_ = 12*λ*_0_ and *x*_1_ = 20*λ*_0_, attached directly to a plastic substrate layer of thickness *L*_1_ = 2.0*λ*_0_. We use weakly ionized gold (Au) and polystyrene plastic (CH) materials with realistic density 19.32 g/cm^3^ and 1.05 g/cm^3^, respectively. The initially ionic charge states of Au, H and C are set to *Z*_*i*_ = 1, 1 and 2, respectively. The corresponding dimensionless ion densities are 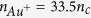 and 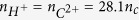.

**Figure 1:**
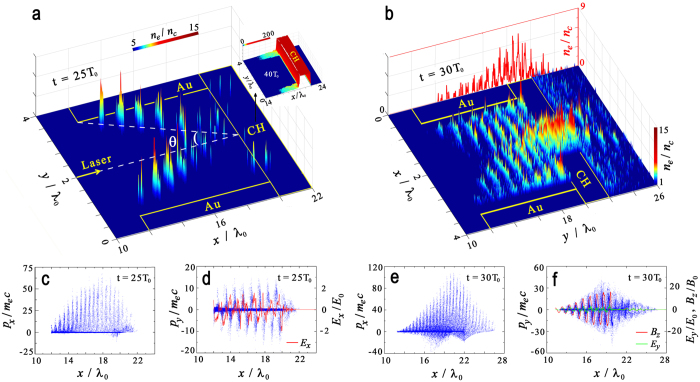
Snapshots of the interaction at *t* = 25*T*_0_ and 30*T*_0_ showing the density of the Au-electron in the channel [(**a**) and (**b**)], the longitudinal [(**c**) and (**e**)] and transverse [(**d**) and (**f**)] momenta for the CT. Here, *θ* in panel (**a**) represents the angle between the electron trajectory and the laser axis, and the inset is the distribution of the CH-electron density; The flank in panel (**b**) plots the on-axis Au-electron density along the laser propagation direction; The red line in panel (**d**) gives the longitudinal electric-field *E*_*x*_ along the *y* = 3.4*λ*_0_ direction excited in the channel. The lines in panel (**f**) are the on-axis magnetic-(*B*_*z*_, red line) and electric-(*E*_*y*_, green line) fields in the channel. *E*_0_ = *m*_*e*_*ω*_0_*c*/*e* and *B*_0_ = *m*_*e*_*ω*_0_/*e*.

Figures 1(a) and (b) present the density evolutions of the Au-electrons from the simulation. We can see that two trains of laminar electron pulses separated by a periodic length of ~1*λ*_0_ are dragged out from the channel into the cavity by the laser. This effect can be attributed to breaking of the stimulated Langmuir oscillation for sufficiently large laser amplitudes^27^. Different from the Brunel mechanism^17^, the “dragged-out” electrons are accelerated forward, instead of pushed back into the Au plasma. Meanwhile, owing to the transverse momenta [Fig. 1(d) and (f)], these bunches spread along the lateral direction and partially converge to the central region during propagation inside the channel. They then disperse radially after penetrating through the attached substrate. The electrons of the CH layer move in the opposite direction as a return current to neutralize the positive charged channel walls caused by these ejected electrons, as shown in the inset of Fig. 1(a). The “dragged-out” electrons from the channel are therefore responsible for inducing the rear accelerating field.

We establish a waveguide acceleration (WGA) model to examine the dynamic of the “dragged-out” electrons inside the channel. As the laser propagates through the plasma waveguide channel, the longitudinal electric-field that arises from the waveguide transverse magnetic (TM_*nm*_) mode can be excited to accelerate the electrons with a proper phase (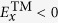)^28^,^29^. In the 2D planar geometry, the channel has an unlimited *z*-direction width (*d*_1_ → ∞). The longitudinal and transverse electric-fields in the channel can be expressed as


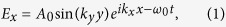



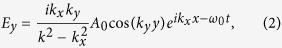


where *A*_0_ is a constant, *k*_*y*_ = *nπ*/*d*_0_, 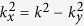, and *k* = 2*π*/*λ*_0_ is the wave number of the incident laser. We can roughly calculate the amplitude of the longitudinal electric-field by 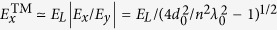. The maximum momentum gains from this field depend on the dephasing time *t*_*d*_ = 2*λ*_0_/(*v*_*ph*_ − *c*) and the acceleration time *t*_*a*_, where 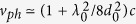 is the laser phase velocity as only the lowest TM_01_ mode is considered for a small *d*_0_. The acquired momentum can therefore be estimated to be 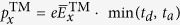, where 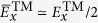 is the averaged accelerating field. For these “dragged-out” electrons, they turn to the forward direction by the *ev*_*y*_*B*_*z*_ force and can be further accelerated by the 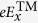 force. Besides, the effect of superluminous phase velocity (*v*_*ph*_ > *c*) in the plasma waveguide channel should also be considered for a wave with its amplitude exceeding the critical value *a*_*cr*_ = [2*c*/(*v*_*ph*_ − *c*)]^1/2^. Following the work^30^ of Robinson *et al*., the electron longitudinal momentum can be rewritten as


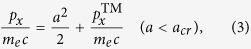



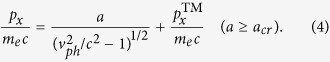


Here, the first terms of the right-hand sides of Eqs (3) and (4) are the contributions from the ponderomotive force, and the second ones denote that of the longitudinal electric-field acceleration. Using *dp*_*y*_/*dt* = *eE*_*y*_ + *ev*_*x*_*B*_*z*_ (essentially, 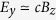 and 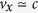), one can immediately obtain the transverse momentum 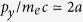. Although *E*_*y*_ almost decreases to zero because the half-wave loss occurs while the laser are reflected by the attached CH-layer at *t* = 30*T*_0_, *B*_*z*_ approaches a 2-fold enhancement due to the change of the light wave-vector direction and thus keep *E*_*y*_ + *cB*_*z*_ constant, as shown in Fig. 1(f). Taking *a* = *a*_0_ and *t*_*a*_ = *τ*_0_, this gives 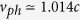, 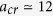, 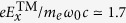, 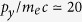 and 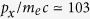. This field amplitude and the momenta show fair consistence with our simulation results in Fig. 1(d)–(f).

**Figure 2:**
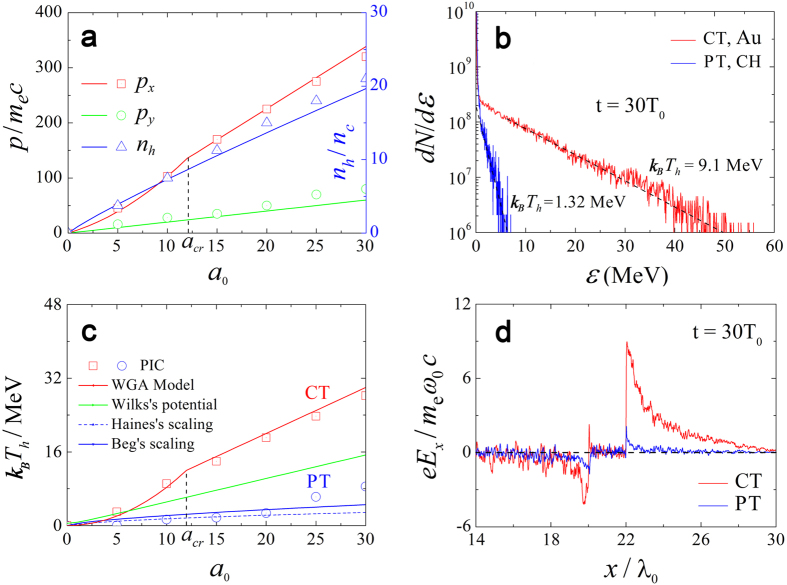
(**a**) The maximum transverse and longitudinal momenta and the highest axial density of the “dragged-out” electrons along the central axis at *t* = 30*T*_0_ versus laser amplitude *a*_0_ for the CT case. (**b**) Spectra of the Au- (CT) and CH- (PT) electrons at *t* = 30*T*_0_ for both cases. The electron temperatures are labeled around the curves. (**c**) The highest Au-electron temperature versus laser amplitude *a*_0_ obtained from the WGA model and PIC simulations. (**d**) The axial profile of the longitudinal electric-field *E*_*x*_ along the axis *y* = 2.0*λ*_0_ at *t* = 30*T*_0_

The maximum electron momenta from Eqs (3)–(4), 14 and 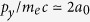 are shown in Fig. 2(a) in a wide laser intensity range, which are in agreement with the simulations. We find that *p*_*x*_ scales as *a*_0_ for *a*_0_ ≥ *a*_*cr*_, which is different from 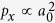 in the case of *a*_0_ < *a*_*cr*_. Due to the fact that 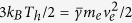, the electron temperature can be simplified to 
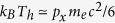
, where 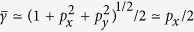 is the averaged electron energy since 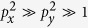 and 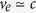 provided the “dragged-out” electrons become fully thermalized. Therefore, we have


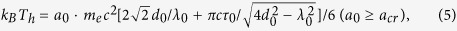


**Figure 3:**
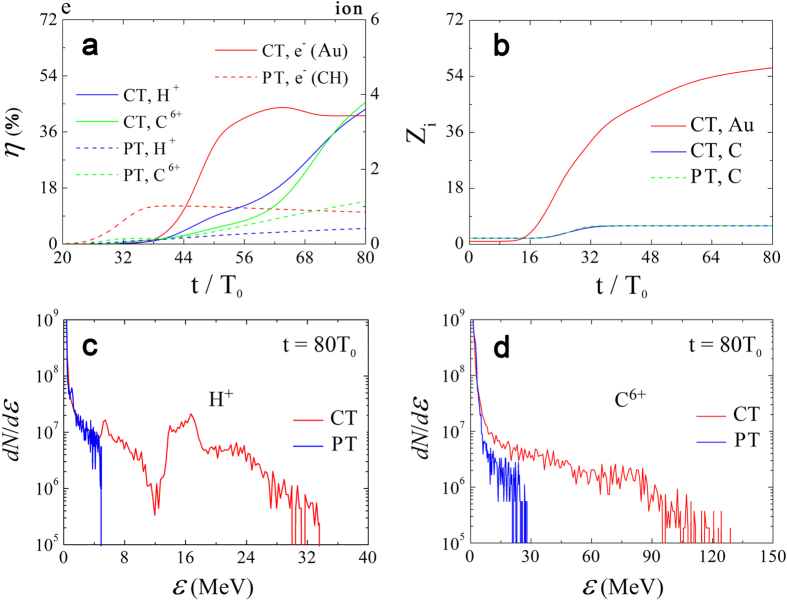
Temporal evolution of (**a**) the energy conversion efficiencies from laser to particles and (**b**) the average ionization degrees of the Au- and carbon-ions. Spectra of (**c**) protons and (**d**) carbon ions at *t* = 80*T*_0_.

Considering 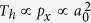 for *a*_0_ < *a*_*cr*_, we obtain


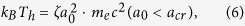


where the proportion coefficient *ζ* can be solved from the continuity of Eqs (5) and (6) at *a*_0_ = *a*_*cr*_. For above given *d*_0_ and *τ*_0_, it is approximated to 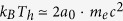 for *a*_0_ ≥ *a*_*cr*_ and 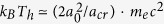, respectively. Taking *a*_0_ = 10, we have 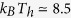 MeV roughly equal to the PIC result 9.1 MeV in Fig. 2(b), which is well above the PT case. Figure 2(c) shows that the theoretical results from Eqs (5)–(6), 32 agree well with the simulations. The temperatures for the CTs are very high, almost twice the Wilks’s ponderomotive potential^1^ 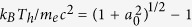. We also notice that the PT results are very low, but in accordance with the Haines’s relativistic model^31^ *k*_*B*_*T*_*h*_/*m*_*e*_*c*^2^ = (1 + 2^1/2^*a*_0_)^1/2^ − 1 and Beg’s experimental fitting^32^ 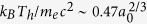. This is due to the fact that the electrons cannot receive all energy from the ponderomotive potential^31^. Further, the sequential ionization of C^6 +^ ions which will reduce the electron temperature is correctly modeled in our simulations. Note that most of the “dragged-out” electron bunches typically have a transverse extent of *l*_*t*_ ~*cT*_0_ = 1*λ*_0_^33^. Due to the charge conservation, assuming that all skin-layer electrons are extracted, the hot-electron density can be estimated to


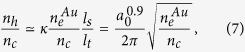


where 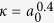 is the ionization modified coefficient^34^, 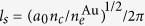 is the skin depth and 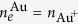. Figure 1(b) [red line] gives that the maximum on-axis electron density at *t* = 30*T*_0_ is as high as ~7.5*n*_*c*_, which is comparable to 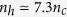 obtained from Eq. (7). As shown in Fig. 2(a) (blue line), the highest density of the “dragged-out” electron bunches shows consistent with the simulations and is far beyond the results observed in other works^18^ (<1*n*_*c*_). The on-axis profile of the quasi-static electric field *E*_*x*_ at *t* = 30*T*_0_ is depicted in Fig. 2(d). Driven by these dense energetic electron bunches, the accelerating electric-field strength is about 36 TV/m high, and the acceleration region is broadened, both of which are very beneficial for the subsequent ion acceleration. Besides, the laser energy is also absorbed more effectively, resulting in a high laser-to-electron conversion efficiency of ~54% in contrast to ~12% of the PT case as shown in Fig. 3(a).

For ultrashort ultra-intense laser pulses, field ionization becomes significant compared to collisional ionization^35^. Figure 3(b) shows that the averaged charge state of the Au ions grows exponentially as soon as the laser impinges on the channel. The final ionization degree approaches 56, consistent with *Z*_*i*_ = 58 calculated by the Ammasov-Delone-Krainov (ADK) model^36^. This corresponds to an extremely high electron density of ~2000*n*_*c*_, which is typically difficult to be modeled using traditional PIC simulations. On the other hand, nearly all carbon-ions are immediately ionized to the 6th ionic charge state due to relatively low ionization threshold. The spectral distributions of protons and C^6 +^ ions at *t* = 80*T*_0_ are presented in Fig. 3(c) and (d). As expected, high energies, up to 33 MeV for protons and 127 MeV for C^6+^ ions, are simultaneously observed. Surprisingly this is almost an order of magnitude larger than that in the planar geometry, where the maximum energy only reaches 5 MeV for protons and 28 MeV for carbon-ions. Consequently, the energy conversion efficiency from laser to ions is increased to ~7.4% from ~1.56% of the PT case as shown in Fig. 3(a). Similar to previously reported results^37^,^38^,^39^,^40^, the protons are accelerated preferentially to heavier carbon-ions. The latter behaves as a buffer to optimize the spectral profiles of the protons, leading to the appearance of the pronounced quasi-monoenergetic peaks with central energies 17 MeV and 1 MeV for both cases, respectively. As a result, the carbon-ion spectra have a typical Maxwellian distribution with cutoff energies.

### Ion energy scaling

**Figure 4:**
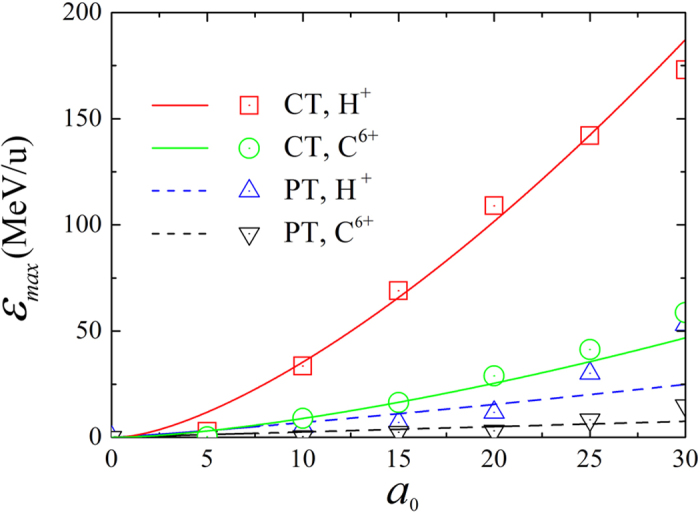
Scaling of the maximum proton and carbon-ion energies at *t* = 80*T*_0_ versus laser amplitude *a*_0_ from the integral of Eq. (8) (lines) and simulations (symbols).

We next extend the laser intensity range to obtain the scaling laws of the maximum energies. According to the model of a two-species plasma expansion^41^, the maximum electric-field beyond the heavy-ion front can be simplified to


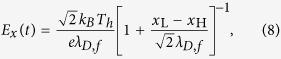


where *λ*_*D,f*_ = (*T*_*h*_/4*πn*_*h*_*e*^2^)^1/2^ is the hot-electron Debye length, and *x*_L_, *x*_H_ are the positions of the light- and heavy-ion fronts, respectively. These can be calculated as 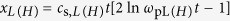, where *c*_*s*,L(H)_ = [*Z*_L(H)_*T*_*h*_/*m*_L(H_)]^1/2^ is the ion acoustic velocity, *Z*_L(H)_ and *m*_L(H)_ are the ionic charge and mass, *ω*_pL(H)_ = [4*πZ*_L(H)_*n*_*e*0,L(H)_*e*^2^/*m*_L(H)_]^1/2^ is the ion plasma frequency, *n*_e0,L(H)_ = *Z*_L(H)_*n*_L(H)_ and *n*_L(H)_ are the initial ion densities, where the subscripts L(H) correspond to the light- (heavy-) ions. By integrating the light-ion equation of motion in this field, we can obtain the maximum light-ion momentum, 
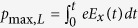
, and energy, *ε*_max_,_L_ = *p*^2^_max_,_L_/2*m*_L_, as a function of time. For the sake of simplicity, the maximum heavy-ion energy is estimated by multiplying a factor 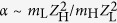 from the hybrid-Boltzmann-Vlasov-Poisson model^39^, i.e., 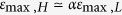. Figure 4 gives the maximum energies of protons and C^6 +^ ions per nucleon in simulations over currently achievable intensities, which grow almost exponentially and show a good agreement with the above analytical model. Furthermore, the results demonstrate that the presence of the metal channel can achieve about 10-fold enhancement of energies for both ion species. With a laser intensity ~10^21^ W/cm^2^ (*a*_0_ = 25), the highest energies are close to 150 MeV for protons and 42 MeV/u (~500 MeV) for C^6+^ ions, which are in the typical energy window of tumor therapy^42^.

### The optimal channel size for the experimental design

**Figure 5:**
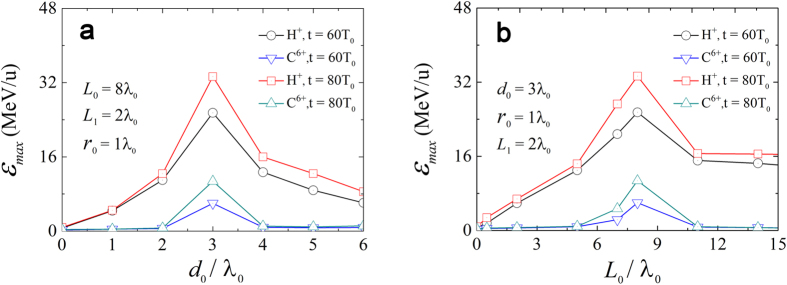
The maximum proton and carbon-ion energies per nucleon as a function of (**a**) the spatial interval *d*_0_ and (**b**) length *L*_0_ at *t* = 60*T*_0_ and *t* = 80*T*_0_ for the CTs, respectively.

To achieve efficient ion acceleration, an important condition is that these “dragged-out” electron bunches can be focused at the central region of the CH-layer front surface. This requires that the angle 
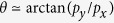
 as illustrated in Fig. 1(a) is comparable to the optimum angle 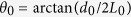. For the above case, 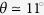 is very close to 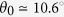, leading to optimal focusing as displayed in Fig. 1(b). The angle matching also provides us with a design method of the optimal channel size, i.e., the relationship 
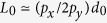
 should be satisfied. The parametric influence of the length *L*_0_ and the spatial interval *d*_0_ on the maximum ion energies is shown in Fig. 5. One note that the maximum energies of protons and C^6 +^ ions are the highest when the spatial interval *d*_0_ is equal to 3*λ*_0_. The reason is that when *d*_0_ is very small, the earlier “dragged-out” electrons blocking the channel entrance prevent the laser from propagating into the channel; conversely if *d*_0_ is too large, it is difficult for these electron bunches to focus at the CH-layer front surface. Different from the nanostructured-attached targets, where surface plasmon resonance excitation^43^ or multipass stochastic heating^44^ may be excited to strength the laser absorption, a proper channel length is essential in our proposed mechanism. If the length of the channel is too short, the laser cannot throw up enough “dragged-out” electron bunches since they have a typical spacing of 1*λ*_0_; whereas if it is too long, early defocusing will weaken the subsequent acceleration. There therefore exists an optimal length *L*_0_ = 8.0*λ*_0_ as seen in Fig. 5(b), which is in accordance with the above predicted 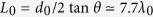.

## Discussion

In the past decade, a large amount of efforts have been dedicated to the research into the boost of ion beam energies, such as using undersense or near-critical density plasmas^19^,^20^,^45^, porous-structured-films^21^,^22^,^23^, and micro-tubes^25^,^26^. Most of these schemes are based on the improvement of the hot electron temperature. For instance, the direct laser acceleration (DLA)^46^,^47^ in the undersense plasmas is a very effective mechanism to heat the electrons and then improve the ion energies. In the DLA mechanism, the electron temperature scales as the laser amplitude^46^, i.e., *T*_*h*_ ~ 1.5*a*_0_, which is comparable to our scheme. However, it is known that the sheath electric-field in TNSA is proportional to the square root of *n*_*h*_*T*_*h*_, and simultaneously great increase of *n*_*h*_ and *T*_*h*_ is therefore vital to significantly increase the ion energies. Benefiting from high-density high-temperature electron bunches dragged from the channel, the energy gain in our scheme is much higher than that in these enhanced TNSA schemes^21^,^22^,^23^,^45^,^48^ based on the DLA and other methods. As a result, the total laser energy (only 1.2 J in our scheme) is far below ~50 J in the previous works^45^,^48^ to generate multi-100 MeV ions. Besides, the direct laser acceleration (DLA) of the electrons essentially depends on the laser pulse propagating in infinitely homogeneous plasmas. In contrast, the mode conversion from the electromagnetic (EM) mode of the laser to the transverse magnetic (TM) mode occurs in the case of laser propagating in a waveguide channel. A longitudinal electric field of the EM mode is excited in the channel, which plays a crucial role in the acceleration.

Here, plasma micro-channel target is used to provide the overdense hot-electron bunches with “superponderomotive” temperatures. After being accurately focused with a proper channel size, they penetrate through the attached plastic substrate to induce a strong sheath electric-field. Compared with the usual planar targets, a ~10 times energy boost of protons and carbon ions is achieved. The optimal channel size and the ion energy scaling are obtained from the analytical model and are confirmed by simulations. This method offers possibilities to obtain hundreds of MeV proton and carbon-ion beams suitable with the present laser facilities.

## Methods

### Numerical simulations

For high-Z target materials, PIC simulations in previous works generally set an averaged ionization degree, forming a fixed electron density for simplicity; moreover, reduced target density is also assumed owing to limited computational resources. One note that the highest electron density reaches 
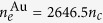
 and 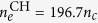 for completely ionized Au and CH materials, which is far beyond the capability of the available high performance computers. To solve this problem, a Voronoi particle merging algorithm^49^ has been implemented in the code VLPL^50^, which effectively controls the excessively increasing simulated particle numbers due to the sequential ionization. In our simulation, the simulation box is *x* × *y* = 48*λ*_0_ × 4*λ*_0_ with a cell size of 0.02*λ*_0_ × 0.02*λ*_0_ and a time step Δ*t* = 0.002*T*_0_. We use 32 macroparticles per cell and initially cold plasmas. For both particles and fields, we used the periodic boundary conditions.

## Additional Information

**How to cite this article:** Zou, D. B. *et al*. Laser-Driven Ion Acceleration from Plasma Micro-Channel Targets. *Sci. Rep.* **7**, 42666; doi: 10.1038/srep42666 (2017).

**Publisher's note:** Springer Nature remains neutral with regard to jurisdictional claims in published maps and institutional affiliations.
