# Erratum: Laser-Driven Ion Acceleration from Plasma Micro-Channel Targets

**DOI:** 10.1038/srep44956

**Published:** 2017-03-23

**Authors:** D. B. Zou, A. Pukhov, L. Q. Yi, H. B. Zhuo, T. P. Yu, Y. Yin, F. Q. Shao

Scientific Reports 7: Article number: 4266610.1038/srep42666; published online: 02 20 2017; updated: 03 23 2017

The original version of this Article contained a typographical error in the spelling of the author H. B. Zhuo, which was incorrectly given as H. B. Zhou. This has now been corrected in the PDF and HTML versions of the Article.

